# Nonsurgical management of periapical lesions

**DOI:** 10.4103/0972-0707.73384

**Published:** 2010

**Authors:** Marina Fernandes, Ida de Ataide

**Affiliations:** Department of Conservative Dentistry and Endodontics, Goa Dental College and Hospital, Bambolim, Goa - 403 601, India

**Keywords:** Calcium hydroxide, cyst, decompression, healing, granuloma, periapical

## Abstract

Periapical lesions develop as sequelae to pulp disease. They often occur without any episode of acute pain and are discovered on routine radiographic examination. The incidence of cysts within periapical lesions varies between 6 and 55%. The occurrence of periapical granulomas ranges between 9.3 and 87.1%, and of abscesses between 28.7 and 70.07%. It is accepted that all inflammatory periapical lesions should be initially treated with conservative nonsurgical procedures. Studies have reported a success rate of up to 85% after endodontic treatment of teeth with periapical lesions. A review of literature was performed by using electronic and hand searching methods for the nonsurgical management of periapical lesions. Various methods can be used in the nonsurgical management of periapical lesions: the conservative root canal treatment, decompression technique, active nonsurgical decompression technique, aspiration-irrigation technique, method using calcium hydroxide, Lesion Sterilization and Repair Therapy, and the Apexum procedure. Monitoring the healing of periapical lesions is essential through periodic follow-up examinations.

## INTRODUCTION

Bacterial infection of the dental pulp may lead to periapical lesions.[1] They are generally diagnosed either during routine dental radiographic examination or following acute pain in a tooth.[2] Most periapical lesions (>90%) can be classified as dental granulomas, radicular cysts or abscesses.[3,4] The incidence of cysts within periapical lesions varies between 6 and 55%.[5] The occurrence of periapical granulomas ranges between 9.3 and 87.1%, and of abscesses between 28.7 and 70.07%.[6] There is clinical evidence that as the periapical lesions increase in size, the proportion of the radicular cysts increases. However, some large lesions have been shown to be granulomas.[7] The definitve diagnosis of a cyst can be made only by a histological examination. However, a preliminary clinical diagnosis of a periapical cyst can be made based on the following: (a) The periapical lesion is involved with one or more non-vital teeth, (b) the lesion is greater than 200 mm^2^ in size, (c) the lesion is seen radiographically as a circumscribed, well-defined radiolucent area bound by a thin radiopaque line, and (d) it produces a straw-colored fluid upon aspiration or as drainage through an accessed root canal system.[8]

The ultimate goal of endodontic therapy should be to return the involved teeth to a state of health and function without surgical intervention.[9] All inflammatory periapical lesions should be initially treated with conservative nonsurgical procedures.[10] Surgical intervention is recommended only after nonsurgical techniques have failed.[11] Besides, surgery has many drawbacks, which limit its use in the management of periapical lesions.[12,13] Various studies have reported a success rate of up to 85% after endodontic treatment of teeth with periapical lesions.[14–16] A high percentage of 94.4% of complete and partial healing of periapical lesions following nonsurgical endodontic therapy has also been reported.[17]

### Search methodology

An electronic search was conducted in the PubMed database with appropriate MeSH headings and key words related to the nonsurgical management of periapical lesions. A hand search of journals was also conducted to enhance the electronic search results.

### Case selection

The current philosophy in the management of periapical lesions includes the initial use of nonsurgical methods. When this treatment approach is not successful a surgical approach may be adopted.[18] The following factors must be considered, while deciding on the management approach:

#### Diagnosis of the lesion

Many bone destroying lesions closely resemble endodontically related periapical lesions on radiographs. Some of these nonendodontic lesions include ameloblastoma, central fibroma, giant cell lesions, fibrous dysplasia, central hemangioma, primary malignancies, metastatic neoplasms, and inflammatory bone diseases. Teeth related to nonendodontic periapical lesions generally test vital to pulp testing methods. It is essential that the clinician establishes the correct diagnosis to avoid unnecessary treatment of vital healthy teeth.[19,20]

#### Proximity of the periapical lesion to adjacent vital teeth

When the periapical lesion is in close proximity to the apices of vital teeth, adopting a surgical approach may result in injury to the blood vessels and nerves of the adjacent teeth, thereby compromising their vitality.[12,13]

#### Encroachment on anatomical structures

Surgery increases the risk of damage to the anatomic structures such as mental foramen, inferior alveolar nerve and / or artery, nasal cavity and maxillary sinus.[19] Also, the aspiration–irrigation technique, a nonsurgical method, is not recommended where adjacent tissue spaces or sinus cavities are involved.[18] In such cases, alternative nonsurgical methods can be used.

#### Patient cooperation

Considerable pain or discomfort can be experienced by the patient during or after a surgical procedure. A nonsurgical approach would be recommended for apprehensive and uncooperative patients.[19] However, patient cooperation is also essential, while using the nonsurgical methods as several follow-up appointments may be required.

#### Age of the patient

Very old patients may not tolerate surgical procedures well and hence may require nonsurgical treatment modalities.[19]

#### Obstructions in the root canal system

Ledges, calcified canals, separated instruments may prevent access to the apical foramen and may warrant a surgical approach in managing periapical lesions related to such teeth.[21]

#### Time involved for treatment

Enhanced healing kinetics are observed after performing apical surgery in teeth with periapical lesions.[22] Although the surgery has many pitfalls, it may be advisable in cases when the patient will be lost to follow-up before complete healing.[11]

#### Cases refractory to nonsurgical management methods

Inflammatory apical true cysts and the presence of cholesterol crystals have been suggested as possible causes that prevent healing of periapical lesions.[23] Surgery is recommended for such cases that do not respond favorably to nonsurgical methods of treatment.[11]

## METHODS FOR NONSURGICAL MANAGMENT OF PERIAPICAL LESIONS

### Conservative root canal treatment without adjunctive therapy

Bhaskar has suggested that instrumentation should be carried 1 mm beyond the apical foramen when a periapical lesion is evident on a radiograph. This may cause transitory inflammation and ulceration of the epithelial lining resulting in resolution of the cyst.[24] Bender in his commentary on Bhaskar’s hypothesis has added that penetration of the apical area to the center of the radiolucency establishes drainage and relieves pressure. Once the drainage stops, fibroblasts begin to proliferate and deposit collagen; this compresses the capillary network, and the epithelial cells are thus starved, undergo degeneration, and are engulfed by the macrophages.[25] Although this proves to be an effective method Shah suggests the possibility that quiescent epithelial cells may be stimulated by instrumentation in the apical region, with resultant proliferation and cyst formation, and thus stressed on the need for follow-up for a period of at least two years.[16] Healing of large cysts like well-defined radiolucencies following conservative root canal treatment has been reported. Although the cystic fluid contains cholesterol crystals, weekly debridement and drying of the canals over a period of two to three weeks, followed by obturation has led to a complete resolution of lesions by 12 to 15 months.[26]

### Decompression technique

The decompression technique involves placement of a drain into the lesion, regular irrigation, periodic length adjustment, and maintenance of the drain, for various periods of time.[27] The drain could either be ‘I’ shaped pieces of rubber dam,[28] polyethylene tube along with a stent,[29] hollow tubes,[30,31] a polyvinyl tubing,[27] suction catheter[32] or a radiopaque latex tubing.[33] There is no standard protocol as to the length of time necessary to leave the drain. It may be different for different kinds, sizes or locations of lesions.[33] It can vary between two days[27] to five years.[32] Daily irrigation of the lesion can be carried out by the patient through the lumen of the drain using 0.12% chlorhexidine.[33,34] The advantages of this technique are; it is a simple procedure, it minimizes the risk of damaging adjacent vital structures, and is easily tolerated by the patient.[27] However, several disadvantages have also been noted; patient compliance is very essential, inflammation of the alveolar mucosa, persistence of the surgical defect at site, development of an acute or chronic infection, displacement or submergence of the drainage tube.[35,36] Rees suggests placement of a small amount of red wax over the end of the drain to prevent ulceration of the labial or buccal mucosa adjacent to the drain.[32] The decompression technique is contraindicated in cases of large dental granulomas or any solid cellular lesion, assince there is an absence of a fluid-filled cavity to decompress.[27]

### Active nonsurgical decompression technique

This technique uses the Endo-eze vacuum system (Ultradent, Salt Lake, Utah) to create a negative pressure, which results in the decompression of large periapical lesions. The high-volume suction aspirator is connected to a micro 22-gauge needle, which is inserted in the root canal and activated for 20 minutes, creating a negative pressure, which results in aspiration of the exudate. When the drainage partially stops, the access cavity is closed with temporary cement, which helps in maintaining bacterial control. Unlike the decompression technique, this technique is minimally invasive as the entire procedure is done through the root canal and causes less discomfort for the patient.[36]

### Aspiration and irrigation technique

Hoen *et al*, suggested aspiration of the cystic fluid from the periapcial lesion using a buccal palatal approach. In this technique, an 18-gauge needle attached to a 20 ml syringe is used to penetrate the buccal mucosa and aspirate the cystic fluid. A second syringe filled with saline is then used to rinse the bony lesion. The new needle is inserted through the buccal wound and passed out through the palatal tissue creating a pathway for the escape of the irrigant.[18] Accumulation of cystic fluid within a confined bony cavity leads to increased hydrostatic pressure, which causes additional osteoclastic activity and growth of the cyst.[37,38] Aspiration leads to decreased hydrostatic pressure, which slows the osteoclastic activity and enlargement of the defect. The gentle irrigation cleanses the bony defect and initiates bleeding and subsequent clot formation, which could be the start of the healing mechanism.[18] The disadvantage of this technique is the creation of buccal and palatal wounds that may cause discomfort to the patient.[39]

#### Aspiration through the root canal technique

To overcome the disadvantage of the traditional aspiration–irrigation technique, a simple technique of aspiration through the root canal has been described. In this technique, aspiration of the cystic fluid is done through the root canal by passing the aspirating needle through the apical foramen. This technique eliminates the creation of buccal and palatal wounds, as in the traditional aspiration–irrigation technique. This minimizes the discomfort that the patient may experience. Severely curved canals may limit the use of this technique as the canal anatomy prevents the aspirating needle from reaching the apical foramen. This technique may also not be favorable in narrow-rooted teeth, for example, the mandibular incisors, as the root canal will have to be widened excessively to allow the aspirating needle to pass into the bony cavity, thus weakening the tooth structure.[39]

However, it is advisable not to use either aspiration–irrigation or aspiration through the root canal techniques where adjacent tissue spaces or sinus cavities are involved, when there is no fluid aspiration from the lesion, or in infected periapical lesions.[18,39]

### Method using calcium hydroxide

Calcium hydroxide is a widely used material in endodontic treatment because of its bactericidal effects.[40–44] It is thought to create favorable conditions for periapical repair and stimulate hard tissue formation.[45,46] Souza *et al*,. suggested that the action of calcium hydroxide beyond the apex may be four-fold: (a) anti-inflammatory activity, (b) neutralization of acid products, (c) activation of the alkaline phosphatase, and (d) antibacterial action.[47] A success rate of 80.8[15] and 73.8%[48] has been reported with calcium hydroxide, when used for endodontic treatment of teeth with periapical lesions. It has been suggested that the presence of a cyst may impede or prevent root-end closure of an immature pulpless tooth even with the use of calcium hydroxide.[49] Contrary to this, Çalişkan and Türkün have reported a case in which apical closure and periapical healing have occurred in a large cyst-like periapical lesion following non-surgical endodontic treatment with calcium hydroxide paste and a calcium hydroxide–containing, root-canal sealer.[35] Extrusion of calcium hydroxide beyond the apex was suggested as a factor for the lack of early healing of periapical lesions.[50] However, many investigators advocate that direct contact between calcium hydroxide and the periapical tissues is beneficial for the inductive action of the material.[46,51] A high degree of success has been reported by using calcium hydroxide beyond the apex in cases with large periapical lesions.[15,35,47] It is barium sulphate that is added to the calcium hydroxide paste for radiopacity, which is not readily resorbed when the paste extrudes beyond the apex. However, it has been reported that even though complete resorption of the paste does not occur in some cases, the periapical radiolucency around the paste resolves.[15]

Some studies have reported that long-term exposure of root dentin to intracanal calcium hydroxide leads to a decrease in the fracture resistance of teeth.[52,53] A method using calcium hydroxide, demineralized freeze-dried bone allograft, and Mineral Trioxide Aggregate (MTA) has been described by Chhabra *et al*., for apexification of an immature tooth associated with a large periapical lesion. Calcium hydroxide is used as an antibacterial agent for only 15 days, following which it is irrigated out of the canal using sodium hypochlorite. The demineralized, freeze-dried bone allograft is then packed in the periapical area to form an apical matrix, with the help of finger pluggers. The demineralized bone matrix also acts as an osteoconductive and possibly as an osteoinductive material. MTA is then compacted over the matrix, forming a 5 mm apical plug.[54]

### Lesion sterilization and repair therapy

The Cariology Research Unit of the Niigata University School of Dentistry has developed the concept of ‘Lesion Sterilization and Tissue Repair (LSTR)’ therapy that uses a triple antibiotic paste of ciprofloxacin, metronidazole, and minocycline, for disinfection of oral infectious lesions, including dentinal, pulpal, and periradicular lesions.[55–57] Repair of damaged tissues can be expected if lesions are disinfected.[58] Metronidazole is the first choice because it has a wide antibacterial spectrum against anaerobes.[59] However, some bacteria are resistant to metronidazole, and hence, ciprofloxacin and minocycline are added to the mix.[60] The combination of drugs has been shown to penetrate efficiently through dentine from the prepared root canals especially from the ultrasonically irrigated root canals.[55] The commercially available drugs are powdered and mixed in a ratio of 1:3:3 (3 Mix) and mixed either with macrogol-propylene glycol (3 Mix-MP) or a canal sealer (3 Mix-sealer).[58] A 1:1:1 ratio of the drug combination has also been used.[61] Although the volume of the drugs applied in this therapy is small, care should be taken to check if the patients are sensitive to chemicals or antibiotics.[62] A disadvantage of the triple antibiotic paste is tooth discoloration induced by minocycline. Cefaclor and fosfomycin are proposed as possible alternatives for minocycline, in terms of their antibiotic effectiveness, but further clinical studies are needed to demonstrate their efficacy in the root canal.[63]

### Apexum procedure

Surgically treated periapical lesions show enhanced healing kinetics compared with those treated nonsurgically.[22] Surgical removal of the periapical, chronically inflamed tissue allows a fresh blood clot to form, thereby converting a chronic inflammatory lesion into a new granulation tissue, where healing might proceed much faster.[64,65] The Apexum procedure uses two sequential rotary devices, the Apexum NiTi Ablator and Apexum PGA Ablator (Apexum Ltd, Or Yehuda, Israel), designed to extend beyond the apex and mince the periapical tissues on rotation in a low-speed handpiece, followed by washing out the minced tissue.[66] A clinical trial reported significantly faster periapical healing in the Apexum-treated group (95%) than in the conventional root canal treatment group (39%) at six months, with significantly less postoperative discomfort or pain. However, whether the procedure was able to remove all the periapical inflammatory tissue was beyond the scope of the study conducted. Further studies regarding this procedure are in progress.[22]

### Materials under research

#### Simvastatin

Simvastatin, a hydroxymethylglutaryl-coenzyme A reductase inhibitor, is used as a cholesterol reducing agent that also possess anti-inflammatory activities. Simvastatin has significantly suppressed the progression of induced rat periapical lesions, possibly by diminishing the cysteine-rich 61 (Cyr61) expression in osteoblasts, a potential osteolytic mediator, which in turn suppressed the infiltration of macrophages.[67]

#### Epigallocatechin-3-gallate

Epigallocatechin-3-gallate (EGCG) is a major polyphenol of green tea that has anti- inflammatory properties. EGCG suppressed the progression of apical periodontitis in a rat model, possibly by diminishing Cyr61 expression in osteoblasts and, subsequently, macrophage chemotaxis into the lesions.[68]

#### Assessment of healing of periapical lesions

Repair of periradicular tissues consists of a complex regeneration involving bone, periodontal ligament, and cementum.[69] The area of mineral loss gradually fills with bone and the radiographic density increases.[70] If the cortical plate is perforated, healing begins with the regeneration of the external cortical plate and proceeds from the outside of the lesion toward the inside.[71] Maxillary lesions resolve faster than mandibular lesions due to the presence of a more extensive vascular network in the maxilla, which facilitates resolution. Anterior lesions of both the maxilla and mandible heal at a faster rate than posterior lesions due to the close proximity of the buccal and lingual plates in the anterior segments.[17]

Although clinical as well as radiographic data are used to monitor cases, the relative absence of clinical symptoms in chronic apical periodontitis makes the assessment primarily a radiographic one. Various methods can be used to assess the healing of periapical lesions by interpretation of periodic recall radiographs.[70] The success–failure criteria laid down by Strindberg is primarily a system designed to detect changes in radiographic appearance. The criteria for success are that: (a) the contours, width, and structure of the periodontal margin are normal; (b) the periodontal contours are widened mainly around the excess filling; and the criteria for failure are: (a) a decrease in the periradicular rarefaction; (b) unchanged periradicular rarefaction; (c) an appearance of new rarefaction or an increase in the initial rarefaction.[71] Even though the periapical conditions are viewed as a continuous process of healing or developing periodontitis, the system is strictly dichotomous, that is, there is no middle ground between success and failure.[70] The area measurement assessment method can be used to monitor the healing of periapical lesions. The rate of repair can be calculated by dividing the size differential between the initial and follow-up visits by the number of elapsed months. On the basis of the average healing rate of approximately 3 mm^2^/mo, a 30 mm^2^ lesion will require 10 months for complete resolution. If the lesion becomes larger, remains the same size or demonstrates a below average rate of healing, then surgical intervention must be considered. However, the measurement involves only two dimensions, because it is not possible to evaluate the buccolingual extent.[17] Another assessment tool is the ‘periapical index’ (PAI), which provides an ordinal scale of five scores ranging from ‘healthy’ to ‘severe periodontitis with exacerbating features’.[72] Of late, an ultrasound with color power Doppler has been demonstrated to be an efficacious monitoring tool in the healing of periapical lesions.[73]

At times, scar tissue can develop after conventional endodontic treatment as well as after periapical surgery.[3] If there are no untoward clinical findings, it indicates fibrous healing or healing by scar formation. The radiograph usually shows a trabecular bone pattern radiating from the center that appears as a reduced, but incompletely resolved radiolucency.[74]

Various authors have stressed on the importance of a long observation time for treated teeth with periapical lesions.[14,48,68] In a clinical review by Çalişkan, a follow-up examination ranged from two to ten years.[48] Shah suggested that patients should be recalled at intervals of three months, six months, one year, and two years, to assess the healing of periapical lesions. There is always the possibility that quiescent epithelial cells may be stimulated by instrumentation in the apical region, with resultant proliferation and cyst formation.[16] Hence, follow-up is extremely essential for a period of at least two years.[75]

## CONCLUSION

Nonsurgical management of periapical lesions have shown a high success rate. A nonsurgical approach should always be adopted before resorting to surgery. The decompression and aspiration–irrigation techniques can be used when there is drainage of cystic fluid from the canals. These techniques act by decreasing the hydrostatic pressure within the periapical lesions. When there is no drainage of fluid from the canals, calcium hydroxide or the triple antibiotic paste can prove beneficial. Periodic follow-up examinations are essential and various assessment tools can be used to monitor the healing of periapical lesions. The surgical approach can be adopted for cases refractory to nonsurgical treatment, in obstructed or nonnegotiable canals and for cases where long-term monitoring of periapical lesions is not possible.
